# Two Cultural Models on Infant Motor Development: Middle Class Parents in Israel and the Netherlands

**DOI:** 10.3389/fpsyg.2020.00119

**Published:** 2020-02-05

**Authors:** Ora Oudgenoeg-Paz, Osnat Atun-Einy, Saskia D. M. van Schaik

**Affiliations:** ^1^Department of Pedagogical and Educational Sciences, Faculty of Social Sciences, Utrecht University, Utrecht, Netherlands; ^2^Department of Physical Therapy, Faculty of Social Welfare and Health Sciences, University of Haifa, Haifa, Israel

**Keywords:** infancy, motor development, parental practices, motor habits, parental beliefs

## Abstract

Culture influences the way parents shape children’s environment. Two studies examined cross-cultural differences in parental practices related to motor development in Israel and the Netherlands. In the first study, 198 Dutch and 206 Israeli parents of infants aged 1–7.5 months completed questionnaires measuring parental practices and beliefs regarding motor development. In the second study, 30 Dutch and 30 Israeli parents completed the same questionnaires when their children were 2 and 10 months old. While similarities were found across the cultures, Israeli parents practiced infant prone positioning more. Additionally, Dutch infants spent substantial more time in the playpen. Furthermore, beliefs stressing stimulation and stimulating practices (both more frequent within Israeli parents) predicted better prone skills, shown by the Israeli infants. Findings highlight the diversity of parental practices related to infant motor development.

## Introduction

Ever since the ages and stages of motor development have first been documented, context-specific variation in type and timing of motor development have been reported (Karasik et al., 2015). In particular, motor development in non-Western countries was found to differ from Western norms and these differences were related to differences in parental practices (for an overview see, Cintas, 1995; Adolph et al., 2010; Karasik et al., 2015). Also within the ‘Western world’ cultural variability in the timing of motor milestones attainment is documented (e.g., WHO Multicentre Growth Reference Study Group and de Onis, 2006; De Kegel et al., 2013; Steenis et al., 2015). However, possible factors causing these differences, such as parental beliefs and practices, are not often studied. The few studies that did address this, found that parental beliefs and expectations regarding motor development differ across Western cultures (Hopkins and Westra, 1990; van Beek et al., 2006). More specifically, in a previous study we found clear differences between Dutch and Israeli parents in their parental beliefs about motor development (Van Schaik et al., 2018), such that Israeli parents attributed more importance to stimulation of motor development, following this development in the “right” order and obtaining expert advice. Dutch parents, on the other hand, attributed greater importance to letting children follow their own pace in motor development (Van Schaik et al., 2018). However, these differences in beliefs are presumably mostly relevant for children’s development through their instantiation into parental choices for the childrearing settings or practices. Therefore, in the current study we investigated differences and similarities in the settings of motor development as well as in daily practices that support or restrict motor development in Israel and the Netherlands. Furthermore, we studied whether daily practices are related to parental beliefs and explored whether daily practices and parental beliefs are related to infant motor skills as reported by parents.

The current study used the theoretical framework of the developmental niche to describe the cultural construction of motor development (Super and Harkness, 1986). The developmental niche describes three interacting subsystems shaping and being shaped by the developing infant while acknowledging the dynamic interaction between infants and their context (Super and Harkness, 1986). The first subsystem addresses the physical and social setting. The second subsystem covers parental beliefs about development and parenting, and the third subsystem concerns daily customs and practices of childrearing. Culture is evident in all three subsystems and all subsystems interact with each other and with the developing child (Super and Harkness, 1986).

Following this framework, we would expect that cultural differences in parental beliefs interact to a certain extent with the setting as well as parental practices and ultimately affect infant motor development. We will therefore first test if the differences found in parental beliefs between Dutch and Israeli parents are also found in settings and practices. Second, we will study the relation between parental beliefs, the settings and practices, and infant motor skills. By doing so, we aim to gain more insight into the cultural construction of motor development. Besides its theoretical implications, knowledge about how culture might shape development is possibly also important for practitioners working with families with diverse backgrounds.

The differences in parental beliefs regarding motor development between Dutch and Israeli parents (Van Schaik et al., 2018) are in agreement with previous work showing that parents in the Netherlands held strong beliefs about the importance of rest and regular routines for infants (Harkness and Super, 2006; Harkness et al., 2007). To the best of our knowledge, no research has studied Israeli views on rest and regularity or activation in infancy. Empirical evidence does show that while the Israeli culture is seen as the least individualistic among Western cultures, Israeli parents are often child-centered and stress the acquisition of autonomy and self-expression (Hofstede, 1983; Bornstein et al., 1998; Feldman et al., 2006).

### The Settings of Motor Development

A large body of research has focused on the first subsystem, namely the environment that potentially support or hampers motor development. Multiple studies have focused on differences in motor development, between countries. Focusing on the country as setting in which development occurs, means focusing on the more distal environment. These studies found differences in age of onset of motor milestones across countries. For example, normative studies show that motor development of Dutch children is delayed compared to children in other western countries, including Israel (Kohen-Raz, 1968; Shapira and Harel, 1983; Steenis et al., 2015). Studies focusing on more proximal settings, namely specific affordances that could potentially promote motor development, show that providing space, objects, and equipment can promote motor development (e.g., Abbott and Bartlett, 2001; Müller et al., 2017).

Cross-cultural differences in the setting of early development might be attributed also to environmental aspects such as climate (e.g., Cintas, 1995; Atun-Einy et al., 2013) or housing conditions (Ammar et al., 2013). Moreover, cross-cultural differences exist in the way parents choose to shape children’s environment. For example, some cultures create settings that enable and even vigorously direct early sitting, while others avoid early sitting altogether (Keller et al., 2002; Karasik et al., 2015). Characteristics such as climate, housing, and landscape might influence parental choices regarding for example clothing, play surfaces, and means of transportation.

The two cultural contexts included in the current study show both similarities and differences in aspects of the setting that might be relevant for early motor development. The weather in Israel is characterized by dry, hot summers and moderate semi-rainy winters. The Netherlands has cool rainy summers and moderate rainy winters. The countries also differ in their topography. The Netherlands is flat with many water sources while Israel has more mountains and hills. Both countries are densely populated compared to other Western countries (The World Bank, 2018). Israeli families are usually larger than Dutch families (an average of 3.1 versus 1.7 children per family; OECD, 2016), meaning that Dutch homes have on average more space per inhabitant (2 rooms) than Israeli homes (1.1. room per inhabitant^1^). Moreover, both countries hold regulations regarding the access to public parks and playgrounds within both urban and rural areas. In Israel this is defined as at least one park per 15,000 inhabitants in small urban areas to 30,000 inhabitants in larger urban areas (Israeli Government, 2019). In the Netherlands, the government advises that public parks should be accessible to all inhabitants within a maximum of 500 m (Dutch Government, 2019). Additionally, about 30% of the children aged 0–3 years in Israel attend a day care facility, mostly for a full week (Vasen-Sikrun et al., 2016). In the Netherlands, about 56% of the children aged 0–4 years attend day care for, on average 2 days per week (Dutch Central Bureau for Statistics, 2018). Both countries have similar paid maternity leave of 16 weeks and have a free public consulting and screening service for infants^1^. Thus, some differences can be expected in the setting of motor development between the two countries due to these different country characteristics.

### Practices Regarding Motor Development

One of the factors that is assumed to directly cause cultural differences in motor development, is the third subsystem of the developmental niche, namely daily practices and habits of care (e.g., Adolph et al., 2010; Super and Harkness, 2015). This subsystem covers parental choices for the activities they do or avoid doing with their infants, such as placing the infant in specific positions, using specific types of equipment, and attending classes or outdoor activities such as baby swimming. Altogether, there is empirical evidence for a relation between infant motor development and specific daily practices. For example, studies have consistently shown that active positions, such as prone and standing position, have positive effects on motor development, while spending much time in less active positions, such as supine and sitting position, is related to less advanced motor development (e.g., Pin et al., 2007; Lobo and Galloway, 2012; Hewitt et al., 2017). Furthermore, findings regarding the developmental effects of specific baby equipment such as baby walkers or sitting devices vary, such that some studies find these devices delay motor activity whereas others find no effect (e.g., Abbott and Bartlett, 2001; De Kegel et al., 2013; for a review see, Pin et al., 2007). In addition, practices of constriction such as swaddling have not been shown to affect motor development (van Sleuwen et al., 2007). Last, though less extensively studied, some evidence suggests that participating in classes for baby activities such as baby swimming and baby massage, either as a formal class or as a more general cultural practice, have positive effects on motor development (Hopkins and Westra, 1990; Sigmundsson and Hopkins, 2010; Dias et al., 2013).

While on the one hand empirical work provides some support for the relation between the settings and practices of care and motor development, on the other hand, studies show that motor practices and habits of care vary across cultures (e.g., Adolph et al., 2010; Gomes et al., 2017). For example, practicing standing and sitting, and applying massage or stretching of the limbs is common in African and Caribbean cultures but less common in Western cultures (for reviews see, Adolph et al., 2010; Super and Harkness, 2015). Less is known about differences in such parental practices between Western cultures, that might explain the documented differences in children’s motor skills.

Following the developmental niche framework, the differences in settings, beliefs, and practices are expected to be interrelated and together shape the differences in infant motor development. Given the general differences between Israel and the Netherlands in terms of physical and social setting, and in terms of parental beliefs about motor development, the specific setting of motor development, and parental practices related to motor development are also expected to differ. However, to date, these differences have not been studied. We will study whether the differences found in parental beliefs regarding motor development in Israel and the Netherlands are instantiated in the settings and practices that parents choose in which children’s motor development takes place, and in the link between these beliefs and practices and actual infant motor skills. Therefore, in the current study we will focus on the following three questions: (1) Do the settings and motor practices differ between Dutch and Israeli parents? (2) Is there a relation between beliefs and practices? (3) Are there cross-cultural differences in reported motor skills and are these skills related to beliefs and practices, cross-sectionally and longitudinally? Altogether, this could add to our understanding of the environmental and cultural factors that shape early motor development. Following the literature review, the main aspects of the settings and practices studied included placing of the infant in different positions, activities such as baby swimming and massage, and the physical setting in which infants are placed such as different equipment used with the infants.

As previous empirical work has shown cross-cultural differences in parental beliefs about motor development between Dutch and Israeli parents (Van Schaik et al., 2018) and given the cultural models of rest and regularity in the Netherlands (Harkness et al., 2007) and autonomy and self-expression in Israel (Feldman et al., 2006), some differences in practices and motor skills can be expected. Specifically, it can be expected that Israeli parents will use more practices that stimulate development such as placing the infant in prone position while Dutch parents will engage in less active stimulation. Similarly Israeli parents are expected to design the environment of children in a way that stimulates motor development more. They are expected, for example, to provide the children with more space for free movement and use more stimulating equipment. On the other hand, Dutch parents are expected to place more focus on a restful environment, for example, by swaddling the infant and using equipment such as a playpen.

Although the theoretical model pictures a relation between parental beliefs and parental practices, empirical studies have not always found such a clear link (see for example, Goodnow, 1996; Bornstein, 2002; Wilcox-Herzog, 2002). One reason for the lack of clear links between beliefs and practices might be because such a link is only found when both are studied at the same level of specificity (Ajzen, 2005). This idea was supported by a study among early education teachers, where a relation was not found between general beliefs and specific group-supporting practices, but was found between group-centered beliefs and group-supporting practices (Van Schaik et al., 2014). Moreover multiple factors might intervene between beliefs and practices and weaken the link between the two. For example, child factors, parent factors, situational factors and conflicts between beliefs might all cause parents to act differently than suggested by the beliefs they hold based on their implicit cultural model (Harkness et al., 2011). Therefore, finding cultural differences in parental beliefs do not unequivocally attest for cultural differences in actual practices and in child development. The current study will address the relations between beliefs and practices in depth, by focusing on beliefs regarding support of motor development, specific practices supporting motor development and infant motor skills among a cross-sectional and longitudinal sample. Finally, Israeli children are expected to be more advanced motorically compared to Dutch children and beliefs and practices are expected to predict level of motor skills cross-sectionally and longitudinally.

These questions and hypotheses will be analyzed using data from two cross-cultural studies. The first is cross-sectional and involves a large sample. In a previous study (Van Schaik et al., 2018), we investigated parental beliefs of these parents and found clear differences between Dutch and Israeli parents. In this study, we will focus on parental practices, settings and children’s skills. The second study is an exploratory small-scale longitudinal study, in which we will focus on the longitudinal relation between beliefs, settings, practices, and children’s skills.

## Materials and Methods

### Participants and Design

The sample of study 1 included 404 parents (198 Dutch and 206 Israeli) of first-born children between 1 and 7.5 months old (*M* = 4.03, *SD* = 1.46, 48.5% girls). Parental age ranged from 21 to 48 years (*M* = 30.28 *SD* = 3.92), most participants (96%) were mothers and 78.5% of the parents had completed university (i.e., obtained at least a bachelor degree). In study 2, 60 mothers (30 Dutch and 30 Israeli) of first-born children participated when the children were about 2 months old (*M* = 2.90, *SD* = 1.20) and again when the children were about 10 months old (*M* = 9.51, *SD* = 1.33). Parental age ranged from 24 to 39 years (*M* = 31.23, *SD* = 3.54). Table 1 presents the distribution of background variables in both samples. We chose to include only parents of first-born relatively young infants to minimize the possible effect of development of the infant and of older siblings on parental beliefs and choices in terms of the environment and practices.

**TABLE 1:** Main background variables in the two cultures.

	Study 1		Study 2	
		
	Netherlands	Israel	Netherlands	Israel
Child age	*M* = 3.95	*M* = 4.11	*M* = 3.13	*M* = 2.67
measurement 1	*SD* = 1.37	*SD* = 1.54	*SD* = 1.46	*SD* = 0.82
Child age	NA	NA	*M* = 9.72	*M* = 9.30
measurement 2			*SD* = 0.57	*SD* = 1.79
Mother’s age	*M* = 30.01	*M* = 30.52	*M* = 31.23	*M* = 32.10
	*SD* = 3.91	*SD* = 3.93	*SD* = 3.54	*SD* = 4.23
Birth weight	*M* = 3425.01 g	*M* = 3313.64 g	*M* = 3647.07 g	*M* = 3225.50 g
	*SD* = 519.81	*SD* = 712.11	*SD* = 910.71	*SD* = 454.83
Child’s gender	49% female	48% female	50% female	47% female
Percentage of children who received physiotherapy	14%	13%	33.5%	20%
Educational level^a^	63% university, 31%	93% university, 1.5%	87% university	100%
	vocational, 6%	vocational, 5.5%	13%	university
	secondary school	secondary school	vocational	

*^*a*^Educational level was determined based on highest level of completed education divided in three levels: secondary school or lower; higher vocational education; and university education.*

### Procedure

This study was approved by the Ethics Committee of the faculty of social sciences at Utrecht University, as well as by the Ethics Committee of Haifa University. Participants were recruited by posting adds around the university and other central locations, on social media, through the researchers’ personal network and using the snow-ball method. All participants provided written informed consent. All infants were born full-term without developmental or medical problems. The questionnaires were administered online using FormLogix in Israel and Limesurvey in the Netherlands. Each questionnaire took about 15 min to complete. In study 2, parents filled in the questionnaires at both measurement moments. Parents received a small gift for their children at time 1 and a gift voucher at time 2.

### Measures

#### Parental Beliefs About Motor Development

The Parental Beliefs on Motor Development (PB-MD) questionnaire (Atun-Einy et al., 2017) was used. The PB-MD consists of five scales measuring the beliefs that: (1) stimulation of motor development is important; (2) motor development occurs naturally; (3) seeking advice on motor development is important; (4) order of motor development is important; and (5) children should follow their own pace in motor development. The items used included general statements regarding motor development (e.g., ‘In typically developing infants, motor development occurs naturally and there is no need to actively stimulate it.’) and vignettes describing realistic scenarios regarding motor development (e.g., “Amy is a 4-month-old girl. She doesn’t like to be on her belly and cries and fusses. Her mother puts her on her back…”) followed by statements representing a continuum of approaches from active (e.g., “the mother must put her baby on her belly even if she cries”) to less active (e.g., “the mother should not worry nor force her baby to be on her belly”). The parents rated their agreement to the statements on a six-point scale ranging from 1 (disagree) to 6 (strongly agree). In line with Atun-Einy et al. (2017), scale scores are the mean of all items within a scale. Additionally, parents were asked in two open questions about parents’ role in motor development and if parents should do any activities with their infants to promote motor development. For this study we used data from the second question and coded whether parents mentioned putting the infant in (1) prone position, (2) supported sitting, and (3) supported standing. Both the reliability and the validity of the PB-MD were found to be good (Atun-Einy et al., 2017). Furthermore, we have previously tested the measurement invariance of the PB-MD comparing the Dutch and Israeli sample and found sufficient measurement invariance, confirming that parental beliefs can be measured using the PB-MD in both cultures (Van Schaik et al., 2018). Therefore, in the current study, we used the means of all items on each scale as the scale score. All scales had sufficient reliability with Cronbach’s alpha values ranging from 0.68 to 0.75.

#### Parental Practices and Physical Setting

The Motor Habits (MOHAB) questionnaire (Atun-Einy et al., 2017) was used to measure the physical setting, parental practices and the position preference (as an indication of motor skills). The MOHAB questionnaire consists of four sections. In the first section parents were asked to indicate their infant’s level of skill in three positions: prone, sitting, and standing. In the current study this part of the MOHAB was used to indicate motor skills level. For study 1, we focused on skill level in prone position rather than in standing or sitting, as at this age range, this skill is likely to show the most variability. Parents were asked about their child’s level of skill in prone position on four skills: being comfortable in prone position, rolling from back to belly, rolling from belly to back, and belly crawling. Total scores were computed by summing up the skills the infant has attained forming a score range of zero to four. For study 2, we focused on the milestones of crawling on hands and knees and pulling to stand as indicators of motor development at age 10 months, as these milestones show the most variability in this age range in both cultural settings (Shapira and Harel, 1983; Steenis et al., 2015).

In the second section, parents were asked how often they place their infants in different positions. Answers were provided on a four-point scale ranging from never (1) to often (4). Additionally, parents were asked about sleep practices (e.g., sleeping position and use of swaddling).

In the third section, parents were asked about parenting activities such as performing specific exercises and motor activities at home with the infant (e.g., crossing and stretching of the limbs), and participation in classes such as baby swimming. Moreover, parents were asked to indicate if, and if so for how many minutes per day, do they use 11 different types of equipment with their infants (e.g., baby walker, playpen).

Finally, in the fourth section, parents were asked about the physical setting of care including: floor type, layers of clothing, and areas in the house where the infant plays. The choice of activities and settings included in the MOHAB was based on an extensive literature review, followed by pilot testing and observations used to identify the prevalent and relevant aspects to be included in the questionnaire. Face and convergent validity of the MOHAB were found to be good (Atun-Einy et al., 2017).

### Data Analysis

Analyses were conducted in SPSS^®^ Version 24 and Mplus version 8.0 (Muthén and Muthén, 1998–2017). In both studies, sample differences in the settings, practices, and beliefs were tested using χ^2^ tests, regression analysis, and MANOVA. The relations between beliefs and practices within the first study were analyzed using multigroup structural equation modeling (Kline, 2005). For this analysis missing data were dealt with by using full information maximum likelihood estimation in Mplus (Muthén and Muthén, 1998–2017). For all other analyses listwise deletion was used. The relations between beliefs, practices, and infant motor skills (based on infants’ level of skill and position preference as described in the MOHAB) were tested using partial correlations controlling for age (study 1) and logistic regression (study 2). The analyses of equipment use, activities, and preferred positions of the infants in the first study were also conducted for three age groups separately (1–2.5, 2.5–5.5, and 5.5–8 months), as these are expected to change as children grow older. In order to correct for multiple comparisons the false discovery rate control was applied (Benjamini and Hochberg, 1995; Glickman et al., 2014).

## Results

### Descriptive Statistics

All parents completed the PB-MD questionnaire. However, about 9% of the parents (8 Dutch and 30 Israeli) did not compete the MOHAB questionnaire. These parents did not significantly vary from the rest on any of the background variables except level of education in the Israeli sample. Though this difference was significant [χ^2^(2) = 6.85, *p* = 0.033, φ = 0.18], it is minimal, as out of the Israeli parents who did not fill in the MOHAB 90% obtained a university degree compared to 94% in the rest of the Israeli sample. Comparison of the Dutch and Israeli samples on background variables in both studies, revealed no significant differences on most variables except the mean level of parental education, where Israeli parents obtained a university degree more often in study 1 [*t*(402) = 4.81, *p* < 0.001, *d* = 0.49] but not in study 2 [*t*(58) = 0.90, *p* = 0.374]. This difference in educational level is in line with OECD data (OECD, 2014). In addition, in study 2, Dutch infants were significantly heavier at birth (*M*_*NL*_ = 3497.32 g, *SD* = 449.30, *M*_*IL*_ = 3225.50 g, *SD* = 454.83, *t*(56) = 2.29, *p* = 0.028, Cohen’s *d* = 0.60). In Supplementary Tables A,B in the Supplementary Material correlations between all measured practices, educational level, and birth weight are presented for both studies. The correlations show that educational level does not correlate with the reported practices. Birth weight is also generally not related to the practices parents used. However, few significant correlations were found only in the Israeli sample suggesting that parents of infants with higher birth weights tend to put them more on the floor surface, less in the playpen, and attend baby swimming less often. Nevertheless, a series of *t*-tests showed that birth weight is not related to any of the outcome variables of infant’s motor skills at age 2 and 10 months. Overall, the outcomes of the study are not likely to be caused by the differences in educational level or birth weight. Therefore, these variables were not included in further analyses.

### Question 1: Cross-Cultural Comparison of Settings and Practices

#### Settings

The first research question concerned cross-cultural differences in the setting of motor development. Results (using data from study 1) show cross-cultural similarities and differences in where infants play and with what equipment. The descriptive and sample difference statistics can be found in Table 2. These results show that a vast majority of the Israeli infants play in the living room directly on the (often tiled) floor surface or on some type of underlying surface. About a third of infants also play in the baby’s room, on the sofa in the living room or outside. Similarly, in the Netherlands most infants also play in the living room, but, compared to the Israeli infants, they more often play in the playpen or on the sofa. Dutch infants also play on the (often laminate or wooden) floor surface but to a lesser extent than Israeli infants. When asleep, a small group of Israeli infants is swaddled and only few are reported to sleep in a sleeping bag. On the contrary, the vast majority of Dutch infants sleep in a sleeping bag. Similar to the Israeli infants, only a small group is swaddled during sleep. Infants in Israel were reported to wear slightly more layers of clothing indoors (though not outdoors) than Dutch infants. All differences were also analyzed for three separate age groups (see Supplementary Table C in Supplementary Material), showing relative stability across age mostly for the large effects.

**TABLE 2:** Settings and practices.

	Netherlands	Israel	χ^2^(*df*)	φ
	**Study 1 – Chi square tests**			

Let infant play in the living room	98%	97%	0.27(1)	0.03
Let the infant play in the baby’s room	14%	38%	78.27(2)***	0.47
In the living room, let the infant play on:				
(1) Floor surface (mat, carpet, or floor)	74.7%	90.7%	15.05(1)***	0.21
(2) Sofa	42%	30%	5.76(1)*	0.13
(3) Playpen	92%	16%	211.54(1)***	0.76
(4) Directly on the floor	20%	3%	23.78(1)***	0.26
Type of floor			172.17(6)***	0.70
(1) Carpet	11%	4%		
(2) Wood	24%	7%		
(3) Laminate	42%	0%		
(4) Tiles	15%	77%		
(5) Linoleum	2%	0%		
Playing outside	11%	27.5%	16.18(1)***	0.21
Cycling	2%	0%	NA	NA
Sleeping outside	2%	2%	NA	NA
Baby swing on the playground	0%	3%	NA	NA
Swaddling during sleep	17%	19%	0.21(1)	0.02
Use of sleeping bag	73%	4%	181.41(1)***	0.71

	**Study 2– Chi square tests**			

Use of sleeping bag	96.5%	0.03%	52.27(1)***	0.93
In the living room, let the infant play on:				
Floor surface (mat, carpet, or floor)	90%	93%	0.22(1)	0.06

	**Study 1 MANOVA**			

			***F*(df)**	$\eta_{\text{p}}^{2}$
			
Multivariate results			22.95(2,344)***	0.12
Layers of clothing indoors	*M* = 2.32 *SD* = 0.53	*M* = 2.62 *SD* = 0.48	31.66(1,344)***	0.08
Layers of clothing outdoors	*M* = 3.06 *SD* = 0.62	*M* = 3.18 *SD* = 0.66	2.91(1,344)	0.01

Reports of parents about the setting of motor development in both samples.*p < 0.04; ***p < 0.001.*p*-Values were evaluated against an adjusted criterium calculated using the false discovery rate control to correct for multiple comparisons. The correction was jointly performed for data in Tables 2, 3 (including 10 χ^2^ comparisons and two MANOVA’s).

An additional aspect of the setting studied was the use of equipment with the infants. A MANOVA showed cross-cultural differences in the frequency and average duration of equipment use with the infants, when controlling for age [*F*(7) = 11.72, *p* < 0.001, $\eta_{\text{p}}^{2}$ = 0.25]. Univariate results, reported in Table 3, show that in Israel, young infants spent most time in the stroller in a lying position, and further spent their time on an activity mat, in a baby bouncer, the carrier or a car seat. Unlike the Israeli infants, the Dutch young infants spent most time in the playpen. They also spent time on the activity mat and in the baby carrier but less than Israeli infants. Similar to Israeli infants, the Dutch infants spent a lot of time in the stroller in a lying position, some time in a car seat, and in the baby bouncer. Use of a baby walker, jumper, swing, and high-chair were excluded from further analyses as these were mentioned by less than 5 parents or for less than 10 min per day.

**TABLE 3:** Mean time of equipment use.

Equipment	Frequency of use		M(SD) minutes use per day		η^2b^
			
	NL	IL	NL	IL	
	**Age 1–2.5 months (N_NL_ = 33, N_IL_ = 35)**				

Activity mat	45.5%	78%	15.61 (23.91)	36.43 (31.85)	0.12***
Baby bouncer	48.5%	61.5%	15.00 (27.27)	36.88 (81.84)	0.01
Baby carrier	24%	69%	13.48 (38.00)	36.48 (37.92)	0.07***
Car seat	97%	100%	31.21 (24.69)	32.50 (15.93)	0.002
Playpen	97%	19.5%	114.06 (145.58)	17.14 (39.52)	0.08***
Stroller lying	94%	94%	108 (161.83)	103.78 (102.44)	0.00
Stroller sitting	–	3%	–	5.00 (27.39)	0.03**
Baby swing^a^	6%	16%	1.67 (7.36)	6.30 (17.35)	
High chair^a^	33.5%	–	7.58 (14.09)	–	

	**Age 2.5–5.5 months (N_NL_ = 128, N_IL_ = 106)**				

Activity mat	88%	98%	53.94 (50.11)	111.26 (107.31)	
Baby bouncer	79.5%	76.5%	37.80 (39.93)	36.66 (34.87)	
Baby carrier	39.5%	79%	17.41 (50.40)	44.52 (58.39)	
Car seat	93.5%	91%	37.91 (36.72)	34.63 (31.42)	
Playpen	96%	27.5%	96.03 (81.93)	34.89 (101.48)	
Stroller lying	89%	83.5%	62.30(73.37	65.12 (55.48)	
Stroller sitting	5.5%	11.5%	2.13 (9.54)	83.43 (28.84)	
Baby swing^a^	7.1%	18%	2.24 (9.97)	8.73 (26.67)	
High chair^a^	19.5%	1%	5.79 (14.93)	0.05 (0.52)	

	**Age 5.5–8 months (N_NL_ = 29, N_IL_ = 37)**				

Activity mat	79.5%	92%	48.57 (57.30)	137.68 (115.32)	
Baby bouncer	69%	75.5%	26.90 (34.45)	28.39 (32.29)	
Baby carrier	55%	67.5%	13.14 (17.42)	22.14 (27.98)	
Car seat	100%	95.5%	39.89 (46.40)	34.35 (19.85)	
Playpen	87.5%	27%	72.25 (53.71)	17.97 (51.02)	
Stroller lying	63%	43%	31.11 (32.65)	18.71 (36.03)	
Stroller sitting	62%	65.5%	33.83 (35.74)	36.20 (41.06)	
Baby swing^a^	17%	24.5%	1.61 (4.32)	9.59 (20.93)	
High chair^a^	65.5%	22.5%	17.55 (19.34)	9.32 (24.36)	

**p < 0.01; ***p < 0.001. ^*a*^Equipment not included in the MANOVA as it was used for less than 10 min per day. ^*b*^Results of MANCOVA for the entire data set controlled for age. The *p*-values were evaluated against an adjusted criterium calculated using the false discovery rate control to correct for multiple comparisons. The correction was jointly performed for data in Tables 2, 3 (including 10 χ^2^ comparisons and two MANOVA’s).

#### Parental Practices

To test whether parents used different practices across the two cultural contexts, we analyzed the data of study 1. Table 4 presents the descriptive and χ*^2^* comparison statistics of daily practices and attending classes as reported by study 1 parents, divided in three age groups. Most Israeli parents do activities aimed at promoting motor development and almost half of them also use an official method to do this. The vast majority of Israeli infants are often put in prone position, receive massages, and sometimes also stretching and crossing of the limbs is practiced. Most Dutch parents also report doing activities aimed at promoting motor development, but most of them do not use an official method to do so. Unlike Israeli infants, the vast majority of Dutch infants are not often placed in prone position. Like Israeli infants, they also receive massages and sometimes practice stretching and crossing of the limbs, however fewer parents report this than Israeli parents do. In both countries only a small group of parents reports attending baby classes. Dutch infants do attend baby swimming more often as they grow older and sometimes also attend baby massage classes. Israeli infants also attend baby swimming, but fewer infants attend them compared to the Dutch infants.

**TABLE 4:** Parental activities across samples (percentages refer to the number of parents reporting to often engage in this activity).

	Age 1–2.5				Age 2.5–5.5				Age 5.5–8			
			
	NL	IL	X ^2^ (df)	φ	NL	IL	X ^2^ (df)	φ	NL	IL	X ^2^ (df)	φ
Activities to promote motor development	78%	90%	1.61(1)	0.16	91%	88%	0.60(1)	0.05	93%	81%	2.00(1)	0.17
Use of method	12.5%	32%	3.40(1)	0.24	12%	45%	30.27(1)***	0.37	3.5%	50%	16.83(1)***	0.51
Baby massage	44%%	60%	1.64(1)	0.16	55%	64%	1.69(1)	0.09	62%	51.5%	0.76(1)	0.11
Crossing and stretching the limbs	19%	46.5%	5.52(1)*	0.30	42.5%	60.5%	7.09(1)**	0.18	14%	43%	6.68(1)*	0.32
Baby yoga	−	−	NA	NA	−	6%	NA	NA	−	−	NA	NA
Place in prone position^a^			48.28(3)***	0.88	14%	90.5%	125.7(3)***	0.76	38%	89%	35.51(3)***	0.70
Often	3%	90%			14%	89.5%			38%	89%		
Sometimes	9%	0%			21.5%	1%			41.5%	0%		
Seldom	48.5%	6.5%			42.5%	4.5%			20.5%	0%		
Never	39.5%	3.5%			22%	4.5%			0%	11%		
Place in sit position	69%	0%	31.97(1)***	0.72	85%	15%	107.48(1)***	0.70	97%	27%	32.17(1)***	0.70
Place in stand position	22%	3.5%	4,74(1)**	0.28	34.5%	6.5%	24.65(1)***	0.33	58.5%	19%	11.07(1)**	0.41
Baby swimming	12.5%	16,7%	0.22(1)	0.06	28%	5.5%	18.26(1)***	0.29	48.5%	11%	11.83(1)**	0.42
Baby massage class	18%	6.5%	1.88(1)	0.17	30.5%	27.5%	0.22(1)	0.03	22%	43%	3.53(1)	0.23
Music and movement class	−	−	NA	NA	4%	8.5%	NA	NA	3%	21.5%	NA	NA
Baby gym class	−	3%	NA	NA	3%	8.5%	NA	NA	−	−	NA	NA
Baby yoga class	3%	−	NA	NA	−	6.5%	NA	NA	−	5.5%	NA	NA
Developmental course	3%	−	NA	NA	−	17%	NA	NA	−	5.5%	NA	NA

Age 1–2.5 months N NL = 32, IL = 30; Age 2.5–5.5 months N NL = 127 (for classes *N* = 130), IL = 93; Age 5.5–8 months N NL = 29 (for classes *N* = 32), IL = 37; ^*a*^Parents were asked: “When awake, does your baby stay on his belly directly on the floor surface or on a blanket or a mattress?” *p < 0.03; **p < 0.01; ***p < 0.001. The *p*-values were evaluated against an adjusted criterium calculated using the false discovery rate control to correct for multiple comparisons.

Some Israeli infants also attend baby massage classes. A small group of Israeli parents (21 in total) mentioned that they followed a so called ‘developmental course.’ This is an activity group for parents and infants where developmental content is also discussed. Though the specific content can vary, these courses mostly discuss the main developmental issues that occupy parents of young infants, such as motor development, sleep, and feeding.

### Question 2: The Link Between Parental Beliefs and Practices

To examine the relation between parental beliefs and practices, a Multigroup Structural Equation Model was constructed using the main practices among which cross-cultural differences were found as dependent variables. For this analysis, only the data of study 1 was used. For each of these practices we tested whether the five beliefs scales included in the PB-MD predict this practice while controlling for the age of the child, parental education and having seen a physiotherapist. In this multigroup model, two models were fitted, one for each country.

In the first step, all paths in the model were constrained to be equal across the two countries. Over 70% of the Israeli parents reported letting their infant play on the floor surface, therefore a model including this variable failed to converge. Without this variable, the model fit the data well [χ^2^(72) = 64.67, *p* = 0.718, CFI = 1.00, TLI = 1.00, RMSEA < 0.01]. To obtain the most parsimonious model, model trimming was applied by setting all the paths with β and *p*-values lower than 0.10 to0. This resulted in a model with excellent fit indices [χ^2^(88) = 77.07, *p* = 0.791, CFI = 1.00, TLI = 1.00, RMSEA < 0.01].

**FIGURE 1:**
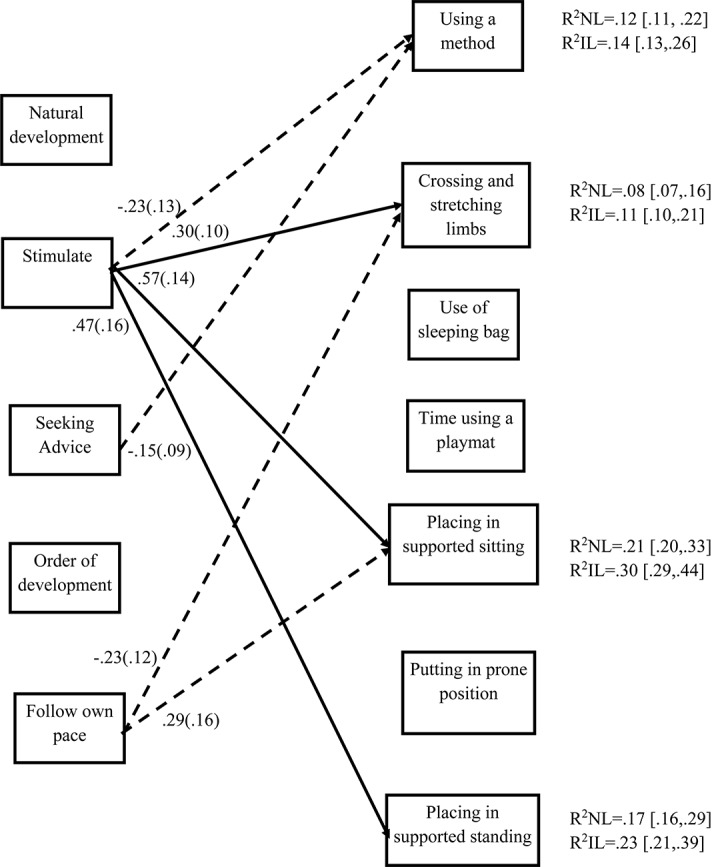
Results of SEM for predictions of practices by beliefs. Only significant paths are presented. Dashed lines are paths significant at the level of *p* < 0.10. Unstandardized path coefficients are reported with the standard error. For simplicity reasons, paths to control variables are not presented. Next to each R^2^-value the confidence interval is reported.

Next, in a stepwise fashion, all the paths between a single practice and beliefs were allowed to vary between the two models, representing the two cultures, one practice at a time. None of these steps delivered a significant improvement in model fit. Therefore, we concluded that the same model could be fitted for both the Israeli and Dutch sample, meaning that the same consistent patterns emerge for the two samples. Figure 1 presents the significant paths that were found in the final model for relations between beliefs and practices and shows that the practices of using a method for the stimulation of motor development, crossing and stretching of the limbs, and placing in sitting and standing position are predicted by parental beliefs. The belief most consistently related to practices is the belief about the importance of stimulation of motor development. Parents who score higher on this belief do more stretching and crossing of the limbs, place their infant more often in supported sitting and standing positions. None of the beliefs predicted the practice of putting the infant in prone position, however this might also be because of low variance in the use of this practice in the Israeli sample (i.e., over 70% of Israeli parents report doing this often).

From the control variables (not presented in Figure 1) age was the most consistent predictor of the practices used. Having attended physiotherapy predicted only the practice of using a method to stimulate motor development and maternal education did not predict any of the practices. The explained variance of each practice by this model ranged from 8 to 30% (see Figure 1), indicating medium to large effect sizes. All of the effect sizes are slightly larger in the Israeli sample. However, the confidence intervals of the effects of the two countries overlap, therefore we cannot conclude that these effects significantly differ.

As noted in the introduction, the link between beliefs and practices should be studied using a similar level of specificity. Therefore, as a next step, we tested whether single items in the PB-MD that specifically relate to beliefs about a certain practice are also statistically related to the use of this specific practice. First, an ANOVA was used to test whether the practice of putting the child in prone position was related to parents’ answers on an item stating that the mother of an infant who fusses in prone position should not worry and not force the baby to be in this position. Results show a significant relation with small to medium effect [*F*(2) = 13.78, *p* < 0.001, $\eta_{\text{p}}^{2}$ = 0.07]. Second, we examined parents’ answers to the open question about their beliefs regarding activities that parents should do with their infants to promote motor development and coded if they mentioned putting the infant in (1) prone position; (2) supported sitting; and (3) supported standing. χ*^2^* test was used to test whether parents’ answers were related to the frequency in which they place their infants in the respective positions. Results reveal that this relation is only significant for putting the infant in prone position [χ^2^(2) = 9.36, *p* = 0.009, φ = 0.17].

### Question 3: Beliefs, Practices, and Reported Infant Motor Skills

#### Motor Skills

As our last research question, the relations between beliefs, practices, and infant motor skills were studied both cross-sectionally and longitudinally. First, using the data of study 1, cross-cultural differences in preferred position and in motor skills were tested. Table 5 reports about infant’s preferred position while awake and during sleep. As this variable represents the infants’ own preference, which may or may not coincide with the position their parents often put them in, it provides an indication for their motor skills. For example, an infant that prefers prone position is probably more competent in this position than an infant who prefers supine position. χ^2^ tests show that, across all ages, Israeli infants spend significantly more time in prone position. Israeli children in both the youngest and oldest age group spend significantly more time being held in arms or the carrier than Dutch children in these age groups. Dutch infants spend more time in supported sitting in all age categories and supported standing in the older age group. Although, in both samples the preferred sleep position shifts with age toward less supine and more prone position, in the Dutch sample this shift is much smaller and significantly more Dutch infants sleep in supine position across all age groups.

**TABLE 5:** Preferred position when awake and during sleep.

	NL				IL					
			
Position when awake:	Never	Seldom	Sometimes	Often	Never	Seldom	Sometimes	Often	X ^2^ (df)	φ
	**Age 1–2.5 months**									

Supine	0%	3%	3%	94%	0%	0%	13.5%	86.5%	3.10(2)	0.22
Prone	15%	18%	48.5%	18%	0%	27.5%	0%	72.5%	29.48(3)***	0.69
Supported sitting	33.5%	12%	33.5%	21%	85.5%	0%	0%	14.5%	20.37(3)***	0.58
Supported standing	82%	12%	3%	3%	96.5%	0%	0%	3.5%	4.62(3)	0.28
Held in arms or carrier	6%	0%	21%	72.5%	3.5%	0%	0%	96.5%	7.41(2)*	0.35

	**Age 2.5–5.5 months**									

Supine	1.5%	1.5%	9.5%	87.5%	0%	2%	11.5%	86%	1.85(3)	0.09
Prone	13.5%	21.5%	46.5%	19%	0%	3%	1%	95.5%	127.45(3)***	0.76
Supported sitting	10%	15%	32.5%	42.5%	42.5%	27.5%	0%	30%	60.51(3)***	0.52
Supported standing	68.5%	10%	16.5%	4.5%	86%	6.5%	0%	7.5%	19.38(3)***	0.30
Held in arms or carrier	7%	5.5%	27.5%	60%	2%	13%	1%	84%	33.77(3)***	0.39

	**Age 5.5–8 months**									

Supine	0%	10.5%	31%	58.5%	2.5%	13.5%	13.5%	70.5%	3.61(3)	0.23
Prone	7%	3.5%	41.5%	48.5%	0%	3%	0%	97%	22.51(3)***	0.59
Supported sitting	7%	3.5%	45%	45%	19.5%	30.5%	3%	47%	21.43(3)***	0.57
Supported standing	41.5%	7%	34.5%	17%	75.5%	11%	0%	13.5%	16.34(3)**	0.50
Held in arms or carrier	0%	7%	55%	38%	0%	13.5%	0%	86.5%	26.97(2)***	0.64

	**Age 1–2.5 months**		**Age 2.5–5.5 months**		**Age 5.5–8 months**					
							
**Sleep position^a^:**	**NL**	**IL**	**NL**	**IL**	**NL**	**IL**				

Supine	93%	39.5%	92.5%	55.5%	92.5%	30.5%				
Prone	3.5%	46.5%	2.5%	33%	7.5%	44.5%				
Side	0%	3.5%	3%	8.5%	0%	19.5%				
Supine and prone	0%	10.5%	0%	1%	0%	0%				
Side and prone	0%	0%	0%	1%	0%	3%				
Side and supine	3.5%	0%	1%	0%	0%	0%				
No preference	0%	0%	1%	1%	0%	3%				
X ^2^ (df)	22.01(4)***		47.94(6)***		25.55(4)***					
φ	0.62		0.47		0.62					

^*a*^Parents reported about a single position as their child’s preferred sleep position. Age 1–2.5 months N NL = 33, IL = 30 (for sleep position N NL = 29, IL = 28); Age 2.5–5.5 months N NL = 127 (for sleep position *N* = 124), IL = 94; Age 5.5–8 months N NL = 29, IL = 37 (for sleep position N NL = 27, IL = 36).*p < 0.033; **p < 0.01; ***p < 0.001. The *p*-values were evaluated against an adjusted criterium calculated using the false discovery rate control to correct for multiple comparisons.

Differences in actual level of motor development as reported by parents were found between the two samples. In study 1 we focused on skill level in prone position. Out of the four skills included in the scale, Israeli infants had on average acquired 1.53 of these skills (*SD* = 1.10) whereas Dutch infants had on average acquired 0.93 of these skills (*SD* = 1.05). This difference entails that Israeli infants were more often comfortable in prone position, and mastered at least one type of rolling whereas Dutch infants usually only attained one type of rolling, and were not yet comfortable in prone position. Regression analysis with age and culture as predictors of number of acquired skills in prone, showed that age is only a marginally significant predictor of number of skills (β = 0.10, *p* = 0.06, *R*^2^ = 0.01) while culture significantly predicts the number of skills and significantly adds to the explained variance of age (β = 0.18, *p* < 0.001, Δ*R*^2^ = 0.03).

### Relations Between Beliefs, Practices, and Motor Skills

#### Cross Sectional Study

Thus, clear differences are seen in children’s preferred positions and levels of motor skill. To test whether these differences can be explained by practices and beliefs, partial correlations between the main practices found to differ between the two cultures, parental beliefs about motor development, and children’s position preference and level of motor skills were calculated while controlling for child’s age. As Table 6 shows, the relations between practices and skill level in prone position are mostly significant with small effect sizes. A few practices stand out as showing no, or very weak relations with prone skill level. These are playing on an activity mat, using a method to promote motor development, crossing and stretching of the limbs, and placing the infant in supported standing position. The practices that show the strongest relations are placing the infant in prone position predicting better skills in prone and placing the infant in supported sitting predicting lower level of skill in prone position. From the beliefs scale, parents who believe the infant should follow its own pace in development tend to have infants with lower prone skills and parents who place significance on order and stimulation of development have infants with better developed motor skills in prone position. Again, effect sizes are small.

**TABLE 6:** Partial correlations controlling for child’s age, between practices, beliefs, and motor skill.

	Skill in prone	Sleep prone	Awake prone
Time activity mat	0.03	0.11	0.17**
Time baby carrier	0.07	0.14*	0.16**
Time playpen	–0.11	−0.15*	−0.15*
Baby swimming	–0.10	–0.08	−0.19**
Playing on the floor surface	–0.03	0.05	0.22***
Using a method	–0.03	–0.09	−0.13*
Cross and stretch limbs	0.11	0.08	0.10
Using sleeping bag	−0.15*	−0.30***	−0.30***
Prone position	0.32***	0.27***	0.44***
Supported sitting	−0.20**	−0.35***	−0.34***
Supported standing	0.00	−0.13*	−0.10^†^
Beliefs:			
Stimulate	0.08	0.10	0.25***
Natural development	–0.02	0.02	–0.03
Seeking advice	0.07	0.06	0.15*
Order	0.23***	0.18	0.31***
Own pace	−0.13*	−0.23***	−0.28***

*p < 0.05; **p < 0.01; ***p < 0.001.

#### Longitudinal Study

To study the relation between beliefs, practices and motor skills longitudinally, an explorative analysis with the data of study 2 (*N* = 60) was conducted. Two logistic regression analyses were performed with dichotomous variables representing the attainment of hands and knees crawling and pulling to stand milestones at age 10 months as dependent variables and beliefs and practices scales as predictors. At 10 months most children had accomplished other milestones such as sitting. Therefore, due to a lack of variance, these milestones were not included. Additionally we controlled for relations between children attending physiotherapy and the outcome variables, as a relatively high percentage of Dutch children in our sample received physiotherapy (see Table 1). Two Chi square analyses revealed no significant relations [χ^2^(1) = 1.07, *p* = 0.301; χ^2^(1) = 0.02, *p* = 0.899]. Therefore, attending physiotherapy was not included in further analyses.

The results of the logistic regression showed that for crawling on hands and knees (obtained by 43% of Dutch and 77% of Israeli infants), a positive relation was found with the seeking advice scale [*b* = 1.14, *p* = 0.014, Exp(B) = 3.12], indicating that an increase of one point on this scale implies that infants are about three times more likely to be crawling at age 10 months. A negative relation was found with the scale of letting children follow their own pace [*b* = −1.11, *p* = 0.031, Exp(B) = 0.33], such that an increase of one point on this scale implies that infants are 0.33 less likely to be crawling at age 10 months. The other beliefs did not predict crawling. Moreover, none of the beliefs significantly predicted the skill of pulling to stand (obtained by 67% of Dutch and 80% of Israeli infants) at age 10 months. Furthermore, no relations were found between practices at age 2 months and motor skills at age 10 months.

## Discussion

The main aim of this study was twofold. First, we aimed to map the cultural models of infant motor development in two Western countries, namely Israel and the Netherlands. Following the developmental niche framework we extended on our previous study reporting differences in parental beliefs about infant motor development (Van Schaik et al., 2018), and set out to test whether beliefs are instantiated into practices and choices of settings. To do this, we studied cross-cultural differences in settings, practices and actual motor development and the link between parental beliefs and practices. Second, as previous work shows clear cultural differences in ages of onset of motor milestones between Dutch and Israeli infants, we wanted to gain further insight in possible explanations for these differences by relating the cultural models (compromised of beliefs, settings and practices) to actual motor development. To reach these aims, the current paper reports on a large cross-sectional and exploratory longitudinal study.

### Cross-Cultural Comparison of the Settings and Practices

Importantly the results show that the differences found in parental beliefs are also found in the setting of motor development. The main differences included the use of an activity mat and placing the infant to play on the floor that were more common in Israel, versus the use of a playpen and a sleeping bag that were more common in Dutch homes. In both countries equipment such as baby walker or swing, that is rather common in North America, was not often used. Also in terms of parental practices clear differences were found between the two samples. The most dramatic difference found was in the practice of placing infants in prone position during daytime and during sleep. This practice was far more common in the Israeli sample. Other main differences found included the practices of placing the infants in supported sitting or standing (more common in the Netherlands), and stretching and crossing of the limbs (more common in Israel). A specific practice mentioned among the Israeli sample, was attending a so called ‘developmental course.’ While the content of these parent–child meetings varies, aspects of (motor) development are also discussed^2^.

Interestingly, the settings and practices that differed between the samples were often either restrictive of movement or extra stimulatory of movement. For example, the use of a sleeping bag and a playpen that were both very prevalent in the Dutch sample, even as children grew older, can be seen as restrictive. A playpen limits the space available for movement, exploration of the environment, and change of location, and does not necessarily elicit prone position. Similarly, a sleeping bag restricts the infant’s movement while in bed. On the other hand, placing the infant to play on the floor surface and placing the infant in prone position, which were both common in the Israeli sample, can be seen as stimulatory of movement. On the floor surface children have more space to move around, explore the environment and change location. While in prone position children can more easily change position by means of rolling or crawling. These findings about the differences in settings and practices that support prone position skills are in line with other studies reporting cross-cultural differences in parental choices for placing children in prone position (Harkness et al., 2007; Carmeli et al., 2008; Zachry and Kitzmann, 2011; Gomes et al., 2017). Moreover, these differences in settings and practices are in line with the hypotheses as they fit with the more general cultural models of both countries. On the one hand the Israeli cultural model emphasizing the acquisition of autonomy and, probably therefore, stimulation of development (Bornstein et al., 1998; Feldman et al., 2006). On the other hand, the Dutch cultural model of supporting rest and regularity during infancy (Harkness and Super, 2006).

### The Link Between Beliefs and Practices

Based on the developmental niche framework (Super and Harkness, 1986) we expected to find explanations for differences in actual motor skills by mapping the two cultural models. In order to find evidence of a cultural construction of motor development, beliefs should be instantiated in practices and both should be related, at least to some degree, to infant development (Harkness et al., 2011). We found several relations between beliefs and practices, though these relations were not very strong. The strongest relations were found between beliefs and practices regarding the support of prone position, in particular when both are measured on equal levels of specificity. Previous studies regarding belief-behavior relations also show that matching level of specificity is important (Ajzen, 2005; Van Schaik et al., 2014). Furthermore, infants whose parents held stronger beliefs about the importance of stimulation and used more practices that support prone position showed higher levels of prone skills. Finding a beliefs-behavior relation regarding specific motor activities, is also in agreement with a recent retrospective study conducted in Brazil showing that the importance parents attributed to specific motor activities (i.e., their attitude) was mostly related to the activities parents did with their infants (Gomes et al., 2017). Taken together, our findings about the differences in the settings and parental practices and the links between beliefs and practices, combined with our previous findings about differences in parental beliefs (Van Schaik et al., 2018), show a system of culturally aligned beliefs, settings and practices. This corresponds with the notion of a heuristic model of parental ethnotheories as presented by Harkness et al. (2011). This cultural system of beliefs and practices is, thus, thought to explain the differences in motor development between the two cultures.

The difference in parental practices regarding prone position when awake and during sleep, is especially interesting because both samples are nested in countries that communicate clear national guidelines regarding the importance of placing the infant in this position as well as sleeping in supine position (Boere-Boonekamp and van der Linden-Kuiper, 2001; Israeli Ministry of Health, 2018). Still, the Dutch parents in our sample support prone position less than the Israeli parents and the Israeli parents let their infants sleep in prone position during daytime naps more often than the Dutch parents. By using the developmental niche framework, further insight into some underlying beliefs that possibly steer parents in their choice of practices was gained. Mostly, the belief that children should follow their own pace could lead parents to stimulate prone position less. On the other hand, beliefs that emphasize the importance of stimulation and order of motor development could lead parents to stimulate prone skills and let children also sleep in this position. Other studies also report that parents possibly have the tendency to ignore recommendations regarding prone position (e.g., Zachry and Kitzmann, 2011). Our finding on prone position placement stress the importance of parental beliefs for both research and clinical practice.

### Beliefs: Settings, Practices, and Actual Motor Skills

As expected, we found cultural differences in prone position skills in both studies, though the effect of culture on these skills in the longitudinal sample was small. Nevertheless, given the multitude of possible influences on motor development (e.g., neurological, biological, genetic), even a small effect of culture is relevant. Moreover, in the cross-sectional study we found a relation between certain practices and prone position skills. This finding was not replicated in the longitudinal sample. This might be due to the relatively small sample size that prevented us from finding small effects. Given the time that has passed between the measurement of the practices and motor skills, only small effects can be expected. Additionally, it could be that to see a relation between practices and skills, measurements should be more proximal in time. Thus, practices at age 2 months, might be too distal from skills measured at age 10 months.

Nevertheless, our findings regarding the differences in prone position skills between the two cultures is in accordance with normative studies showing that Dutch children are somewhat delayed in their motor development compared to Israeli children (Kohen-Raz, 1968; Shapira and Harel, 1983; Steenis et al., 2015). The link found between settings and practices that support prone position skills and these motor skills is agreement with studies showing that regularly placing young infants in prone position while awake provides infants with opportunities to strengthen their upper body and thus facilitate motor development (Majnemer and Barr, 2006; Dudek-Shriber and Zelazny, 2007; Pin et al., 2007) and prevents development of flattening of infants’ heads (positional plagiocephaly; Boere-Boonekamp and van der Linden-Kuiper, 2001). Accordingly, in the Netherlands, relatively higher rates of positional plagiocephaly are reported and this is thought to be related, among other factors, to the low frequency of prone position as found in the current and other studies (Boere-Boonekamp and van der Linden-Kuiper, 2001; van Vlimmeren et al., 2007, 2008). Taken together, our work regarding the difference in prone position skills is in line with previous findings. Moreover, our work adds possible causes of prone position differences and therefore has direct relevance to practitioners that might try to increase prone position placement. Thus, these findings suggest that the system of culturally aligned beliefs, settings and practices shares relations with motor skills.

Our findings suggest that infant motor development indeed is at least partly culturally constructed. This emphasizes the importance of placing infant motor development studies into their ‘cultural cradle.’ In the past few years, most journals have started to require manuscripts to include more elaborate background information about the samples studied, as is mentioned in the APA publication manual (American Psychological Association, 2010). The current study suggest that even more so, besides background characteristics, reports about prevailing cultural models among the participants of a study could give more meaning to research findings, its implications and generalizability (see also Adolph et al., 2010).

### Strengths and Limitations

The current study has a few limitations worth noting. First, we rely solely on parental reports. While parents are the best source of information regarding parenting and child development and parental reports are often used in developmental research, these reports might also be biased. Moreover, as all variables were measured using a survey method, we cannot exclude the possibility of method-bound correlations. However, the types of questions and scaling are very different between the two questionnaires and both questionnaires were found reliable and valid measures of parental beliefs and practices (Atun-Einy et al., 2017). Furthermore, part of the MOHAB entails reporting motor skills, previous studies have shown that this is a reliable method to assess children’s motor development (e.g., Bodnarchuk and Eaton, 2004). Despite the limitations inherent to the use of survey data, the questionnaires enabled us to study a larger group from two cultures.

Conclusions based on the longitudinal data should be drawn with caution, as the sample size was relatively small. Future studies, using large samples, should try to replicate these results. Finally, the sample of the current study was rather homogeneous in terms of SES and gender of the parents, as the current study included mainly middle-class mothers. Future work should include more diverse samples in both sites. Given our sample, this study reflects maternal beliefs and practices rather than parental. While this is not unusual in scientific studies, it is still recommended to include more fathers in future studies. This is especially important as in Western societies the role fathers assume in childrearing is constantly growing (Cabrera et al., 2000). Future work could also include more low-SES parents and alloparents, such as family caregivers or caregivers in child care settings, as infants often spend at least a few days per week being cared for by alloparents (OECD, 2016).

## Conclusion

Future studies could also extend this work by including more qualitative data and analyses regarding beliefs and practices as well as extending the cross-cultural comparison to other cultures and populations. For example, the study of minority populations or parents of children with disabilities. Additionally, future studies could examine specific questions regarding practices that are highly common in one culture but relatively rare in others, more in-depth, such as cycling in the Netherlands or the use of parenting courses such as the ‘developmental course’ in Israel. Through studying the content of these practices more deeply and exploring the motives behind parental choices for certain practices, even more insight can be gained in how these practices shape the context of motor development of children within a culture. Moreover, in the current study we focused mainly on the physical setting of motor development. Future work could investigate the effect of the social setting on motor development.

The current study provided us with a unique opportunity to study both beliefs and practices and study if and how motor development is culturally structured. We found two distinct cultural models that are related to development of infant skills in prone position. This suggests that motor development is, at least partially, culturally constructed. This insight is important for both science and practice. The findings suggest that it might be useful to consider parental beliefs when informing parents about practices such as encouraging prone position (e.g., Harry, 2008; Hewitt et al., 2017). Moreover, these results further underscore the relevance of a cultural approach in studying motor development among Western cultures.

## Data Availability Statement

The datasets generated for this study are available on request to the corresponding author.

## Ethics Statement

The studies involving human participants were reviewed and approved by Ethics Committee Faculty of Social Sciences Utrecht University and Ethics Committee Haifa University. The patients/participants provided their written informed consent to participate in this study.

## Author Contributions

OO-P, SV, and OA-E designed the study. OO-P and SS collected the data in Netherlands. OA-E collected the data in Israel. OO-P analyzed the data and wrote the manuscript. SV and OA-E provided feedback and edited the text.

## Conflict of Interest

The authors declare that the research was conducted in the absence of any commercial or financial relationships that could be construed as a potential conflict of interest.

## Footnotes

1 http://stats.oecd.org/

2 www.first-step.co.il

## References

[B1] Abbott A. L. Bartlett D. J. (2001). Infant motor development and equipment use in the home. *Child Care Health Dev.* 27 295–306. 10.1046/j.1365-2214.2001.00186.x 11350456

[B2] Adolph K. E. Karasik L. B. Tamis-LeMonda C. S. (2010). “Motor skill,” in *Handbook of Cultural Developmental Science*, ed. Bornstein M. H. (New York, NY: Taylor & Francis), 61–88.

[B3] Ajzen I. (2005). *Attitudes, Personality and Behavior.* Milton Keynes: McGraw-Hill.

[B4] American Psychological Association (2010). *Publication Manual of the American Psychological Association*, 6th Edn. Washington, DC: American Psychological Association.

[B5] Ammar D. Acevedo G. A. Cordova A. (2013). Affordances in the home environment for motor development: a cross-cultural study between American and Lebanese children. *Child Dev. Res.* 2013:152094 10.1155/2013/152094

[B6] Atun-Einy O. Cohen D. Samuel M. Scher A. (2013). Season of birth, crawling onset, and motor development in 7-month-old infants. *J. Reprod. Infant Psychol.* 31 342–351. 10.1080/02646838.2013.826347

[B7] Atun-Einy O. Oudgenoeg-Paz O. Van Schaik S. D. M. (2017). Parental beliefs and practices concerning motor development: testing new tools. *Eur. J. Dev. Psychol.* 14 556–604. 10.1080/17405629.2016.1263563

[B8] Benjamini Y. Hochberg Y. (1995). Controlling the false discovery rate: a practical and powerful approach to multiple testing. *J. R. Stat. Soc. Series B Stat. Methodol.* 57 289–300. 10.1111/j.2517-6161.1995.tb02031.x

[B9] Bodnarchuk J. L. Eaton W. O. (2004). Can parent reports be trusted? Validity of daily checklists of gross motor milestone attainment. *J. Appl. Dev. Psychol.* 25 481–490. 10.1016/j.appdev.2004.06.005

[B10] Boere-Boonekamp M. M. van der Linden-Kuiper L. T. (2001). Positional preference: prevalence in infants and follow-up after two years. *Pediatrics* 107 339–343. 10.1542/peds.107.2.339 11158467

[B11] Bornstein M. H. (2002). “Parenting infants,” in *Handbook of Parenting: Children and Parenting*, 2nd Edn, Vol. 1 ed. Bornstein M. H. (Mahwah, NJ: Lawrence Erlbaum Associates), 3–93.

[B12] Bornstein M. H. Haynes O. M. Azuma H. Galperín C. Maital S. Ogino M. (1998). A cross-national study of self-evaluations and attributions in parenting: Argentina, Belgium, France, Israel, Italy, Japan, and the United States. *Dev. Psychol.* 34 662–676. 10.1037/0012-1649.34.4.662 9681258

[B13] Cabrera N. Tamis-LeMonda C. S. Bradley R. H. Hofferth S. Lamb M. E. (2000). Fatherhood in the twenty-first century. *Child Dev.* 71 127–136. 10.1111/1467-8624.00126 10836566

[B14] Carmeli E. Marmur R. Cohen A. Tirosh E. (2008). Preferred sleep position and gross motor achievement in early infancy. *Eur. J. Pediatr.* 168 711–715. 10.1007/s00431-008-0829-4 18795326

[B15] Cintas H. L. (1995). Cross-cultural similarities and differences in development and the impact of parental expectations on motor behavior. *Pediatr. Phys. Ther.* 7 103–111. 10.1097/00001577-199500730-00004

[B16] De Kegel A. Peersman W. Onderbeke K. Baetens T. Dhooge I. Van Waelvelde H. (2013). New reference values must be established for the Alberta infant motor scales for accurate identification of infants at risk for motor developmental delay in Flanders. *Child Care Health Dev.* 39 260–267. 10.1111/j.1365-2214.2012.01384.x 22676145

[B17] Dias J. A. B. Manoel Ede J. Dias R. B. Okazaki V. H. (2013). Pilot study on infant swimming classes and early motor development. *Percept. Mot. Skills* 117 950–955. 10.2466/10.25.PMS.117x30z2 24665810

[B18] Dudek-Shriber L. Zelazny S. (2007). The effects of prone positioning on the quality and acquisition of developmental milestones in four-month-old infants. *Pediatr. Phys. Ther.* 19 48–55. 10.1097/01.pep.0000234963.72945.b1 17304097

[B19] Dutch Central Bureau for Statistics (2018). *Cijfers Kinderopvang Eerste Kwartaal 2018 [Numbers Child Care First Quartile 2018].* Available at: https://www.rijksoverheid.nl/documenten/publicaties/2018/06/25/cijfers-kinderopvang-eerste-kwartaal-2018 (accessed August 20, 2019).

[B20] Dutch Government (2019). *Groene Leefomgeving [Green Environment].* Available at: https://www.atlasleefomgeving.nl/meer-weten/natuur/groene-leefomgeving (accessed December 15, 2019).

[B21] Feldman R. Masalha S. Alony D. (2006). Microregulatory patterns of family interactions: cultural pathways to toddlers’ self-regulation. *J. Fam. Psychol.* 20 614–623. 10.1037/0893-3200.20.4.614 17176196

[B22] Glickman M. E. Rao S. R. Schultz M. R. (2014). False discovery rate control is a recommended alternative to Bonferroni-type adjustments in health studies. *J. Clin. Epidemiol.* 67 850–857. 10.1016/j.jclinepi.2014.03.012 24831050

[B23] Gomes A. M. Ribeiro R. F. Vinolas B. de Castro Magalhães L. (2017). Parental practices and beliefs on motor development in the first year of life. *Fysioter. Mov.* 30 769–779. 10.1590/1980-5918.030.004.AO12

[B24] Goodnow J. J. (1996). “From household practices to parents’ ideas about work and interpersonal relationships,” in *Parents’ Cultural Beliefs System*, eds Harkness S. Super C. M. (New York, NY: The Guilford Press), 313–344.

[B25] Harkness S. Super C. M. (2006). “Themes and variations: parental ethnotheories in western cultures,” in *Parenting Beliefs, Behaviors, and Parent-Child Relations: A Cross Cultural Perspective*, eds Rubin K. H. Chung O. B. (New York, NY: Psychology Press), 61–79.

[B26] Harkness S. Super C. M. Mavridis C. J. (2011). “Parental ethnotheories about children’s socioemotional development,” in *Socioemotional Development in Cultural Context*, eds Chen X. Rubin K. H. (New York, NY: Guilford Press), 73–98.

[B27] Harkness S. Super C. M. Moscardino U. Rha J. Blom M. J. M. Huitrón B. (2007). Cultural models and developmental agendas: implications for arousal and self-regulation in early infancy. *J. Dev. Process.* 5 5–39.

[B28] Harry B. (2008). Collaboration with culturally and linguistically diverse families: ideal versus reality. *Except. Child.* 74 372–388. 10.1177/001440290807400306

[B29] Hewitt L. Stanley R. M. Okely A. D. (2017). Correlates of tummy time in infants aged 0–12 months old: a systematic review. *Infant Behav. Dev.* 49 310–321. 10.1016/j.infbeh.2017.10.001 29096238

[B30] Hofstede G. (1983). National cultures in four dimensions: a research-based theory of cultural differences among nations. *Int. Stud. Manage. Organ.* 13 46–74. 10.1080/00208825.1983.11656358

[B31] Hopkins B. Westra T. (1990). Motor development, maternal expectations, and the role of handling. *Infant Behav. Dev.* 13 117–122. 10.1016/0163-6383(90)90011-V 30165120

[B32] Israeli Government (2019). *Guide for Planning Public Parks.* Available at: https://www.gov.il/BlobFolder/reports/r0969_moch/he/r0969_b.pdf (accessed December 15, 2019).

[B33] Israeli Ministry of Health (2018). *גיל שלושה חודשים עד חצי שנה [Age 3 Till 6 Months].* Available at: https://www.health.gov.il/Subjects/infants/care/guidance_fo_parents_age_of_the_infant/Pages/three_months_to_six_months.aspx (accessed July 1, 2019).

[B34] Karasik L. B. Tamis C. S. Adolph K. E. Bornstein M. H. (2015). Places and postures: a cross-cultural comparison of sitting in 5-month-olds. *J. Cross Cult. Psychol.* 46 1023–1038. 10.1177/0022022115593803 26924852 PMC4767024

[B35] Keller H. Yovsi R. D. Voelker S. (2002). The role of motor stimulation in parental ethnotheories. *J. Cross Cult. Psychol.* 33 398–414. 10.1177/00222102033004003

[B36] Kline R. B. (2005). *Principles and Practice of Structural Equation Modelling.* New York, NY: The Guilford Press.

[B37] Kohen-Raz R. (1968). Mental and motor development of kibbutz, institutionalized, and home-reared infants in Israel. *Child Dev.* 39 489–504. 5652769

[B38] Lobo M. A. Galloway J. C. (2012). Enhanced handling and positioning in early infancy advances development throughout the first year. *Child Dev.* 83 1290–1302. 10.1111/j.1467-8624.2012.01772.x 22540738

[B39] Majnemer A. Barr R. G. (2006). Association between sleep position and early motor development. *J. Pediatr.* 149 623–629.e1. 10.1016/j.jpeds.2006.05.009 17095331

[B40] Müller A. B. Valentini N. C. Bandeira P. F. R. (2017). Affordances in the home environment for motor development: validity and reliability for the use in daycare setting. *Infant Behav. Dev.* 47 138–145. 10.1016/j.infbeh.2017.03.008 28433877

[B41] Muth L. K. Muth B. o. (1998–2017). *Mplus User’s Guide*, 6th Edn. Los Angeles, CA: Muthén & Muthén. 10.1016/j.infbeh.2017.03.008

[B42] OECD (2014). *Education at a Glance 2014: OECD Indicators*. Paris: Organisation for Economic Co-operation and Development 10.1787/eag-2014-en

[B43] OECD (2016). *OECD Family Database*. Paris: Organisation for Economic Co-operation and Development.

[B44] Pin T. Eldridge B. Galea M. P. (2007). A review of the effects of sleep position, play position, and equipment use on motor development in infants. *Dev. Med. Child Neurol.* 49 858–867. 10.1111/j.1469-8749.2007.00858.x 17979866

[B45] Shapira Y. Harel S. (1983). Standardization of the Denver developmental screening test for Israeli children. *Isr. J. Med. Sci.* 19 246–251. 6189803

[B46] Sigmundsson H. Hopkins B. (2010). Baby swimming: exploring the effects of early intervention on subsequent motor abilities. *Child Care Health Dev.* 36 428–430. 10.1111/j.1365-2214.2009.00990.x 19719766

[B47] Steenis L. J. P. Verhoeven M. Hessen D. J. Van Baar A. L. (2015). Performance of Dutch children on the Bayley III: a comparison study of US and Dutch norms. *PLoS One* 10:e0132871. 10.1371/journal.pone.0132871 26267907 PMC4534391

[B48] Super C. M. Harkness S. (1986). The developmental niche: a conceptualization at the interface of child and culture. *Int. J. Behav. Dev.* 9 545–569. 10.1177/016502548600900409

[B49] Super C. M. Harkness S. (2015). “Charting infant development: milestones along the way,” in *Oxford Library of Psychology. The Oxford Handbook of Human Development and Culture: An Interdisciplinary Perspective*, ed. Jensen L. A. (New York, NY: Oxford University Press).

[B50] The World Bank (2018). *Population Density (People Per Sq. Km of Land Area)* Avaliable at: https://data.worldbank.org/indicator/EN.POP.DNST (accessed July 1, 2019).

[B51] van Beek Y. Genta M. L. Costabile A. Sansavini A. (2006). Maternal expectations about infant development of pre-term and full-term infants: a cross-national comparison. *Infant Child Dev.* 15 41–58. 10.1002/icd.428

[B52] Van Schaik S. D. M. Leseman P. P. M. Huijbregts S. K. (2014). Cultural diversity in teachers’ group-centered beliefs and practices in early childcare. *Early Child. Res. Q.* 29 369–377. 10.1016/j.ecresq.2014.04.007

[B53] Van Schaik S. D. M. Oudgenoeg-Paz O. Atun-Einy O. (2018). Cross-cultural differences in parental beliefs about infant motor development: a quantitative and qualitative report of middle-class Israeli and Dutch parents. *Dev. Psychol.* 54 999–1010. 10.1037/dev0000494 29504776

[B54] van Sleuwen B. E. Engelberts A. C. Boere-Boonekamp M. M. Kuis W. Schulpen T. W. L’Hoir M. P. (2007). Swaddling: a systematic review. *Pediatrics* 120 e1097–e1106. 10.1542/peds.2006-2083 17908730

[B55] van Vlimmeren L. A. van der Graaf Y. Boere-Boonekamp M. M. L’Hoir M. P. Helders P. J. M. Engelbert R. H. H. (2007). Risk factors for deformational plagiocephaly at birth and at 7 weeks of age: a prospective cohort study. *Pediatrics* 119 e408–e418. 10.1542/peds.2006-2012 17272603

[B56] van Vlimmeren L. A. van der Graaf Y. Boere-Boonekamp M. M. L’Hoir M. P. Helders P. M. Engelbert R. H. (2008). Effect of pediatric physical therapy on deformational plagiocephaly in children with positional preference: a randomized controlled trial. *Arch. Pediatr. Adolesc. Med.* 162 712–718. 10.1001/archpedi.162.8.712 18678802

[B57] Vasen-Sikrun L. Rotem R. Ben Rabi D. (2016). *שירותים ומדיניותגיל הינקות בישראל:צורכי הילדים וההורים [Infancy in Israel: The Needs of Children and Parents, Services and Policy].* Jerusalem: Myers-JDC-Brookdale Institute.

[B58] WHO Multicentre Growth Reference Study Group, and de Onis, M. (2006). WHO motor development study: windows of achievement for six gross motor development milestones. *Acta Paediatr.* 95 86–95. 10.1111/j.1651-2227.2006.tb02379.x 16817682

[B59] Wilcox-Herzog A. (2002). Is there a link between teachers’ beliefs and behaviors? *Early Educ. Dev.* 13 81–106. 10.1207/s15566935eed1301_5

[B60] Zachry A. H. Kitzmann K. M. (2011). Caregiver awareness of prone play recommendations. *Am. J. Occup. Ther.* 65 101–105. 10.5014/ajot.2011.09100 21309377

