# Supplementary material for: Two Cultural Models on Infant Motor Development: Middle Class Parents in Israel and the Netherlands
Source: Front Psychol. 2020 Feb 5;11:119. doi: 10.3389/fpsyg.2020.00119 (PMC7012936; doi:10.3389/fpsyg.2020.00119)
Supplement: Supplementary file 1 [file Table_1.pdf]

Table A

Correlations between parental practices related to motor development in both cultures, cross-sectional sample.

	1	2	3	4	5	6	7	8	9	10	11	12	13	14	15	16
1. Time activity mat		.07	.04	.02	-.07	-.10	-.03	.08	.03	-.03	.10	.04	-.07	.00	.09	-.04
2. Time baby carrier	.05		-.03	0.04	.002	.02	.04	.08	-.06	.02	-.01	.13	.12	.09	.09	.02
3. Time playpen	.09	-.04		.51***	.28**	.03	.06	.04	.06	-.01	.12	.08	-.04	-.09	-.02	.08
4.Playing on the floor surface	.29***	.10	-.08		-.02	.05	-.004	.04	.05	.25**	-.06	.09	-.12	-.01	.05	.08
5. Playing in the playpen	-.03	.05	.22**	.17*		-.03	-.004	.29***	.13	-.02	.07	.12	.03	.10	-.05	.21**
6. Using a method	-.02	-.09	.09	.13	.03		-.10	.07	.06	-.07	.18*	.05	.05	-.02	-.15	.01
7. Crossing and stretching limbs	.13	.11	-.06	.12	-.01	-.12		.11	.09	-.01	-.05	.04	-.05	.02	.11	.06
8. Placing in sit position	-.05	.06	-.13	.16*	-.01	-.01	.19**		.58**	-.08	.16*	.05	.03	-.17*	-.03	.05
9. Placing in stand position	-.02	.04	-.05	.15*	.003	.08	.02	.33***		-.03	-.06	.07	-.11	-.11	-.01	.15*
10. Baby swimming	.06	-.10	.02	-.10	.09	-.01	-.04	.09	.12		-.07	-.01	-.003	.07	.08	-.02
11. Use of sleeping bag	.18*	.004	.05	.12	.04	-.01	-.05	.001	.05	.15*		-.05	-.07	-.16*	-.07	-.04
12. Frequency of prone position	.21**	-.02	-.08	.50***	.08	-.01	-.001	.16*	.18*	.19**	.30***		.22**	.03	-.09	.01
13. Frequency of being held in arms	-.17*	.19**	-.13	-.06	.10	-.01	.06	-.09	-.02	-.09	-.05	-.05		.07	-.04	-.02
14. Frequency of prone sleeping[a]	-.10	-.05	.04	.06	.00	.10	.07	-.05	.01	.01	-.02	.01	.07		.02	-.10
15. Educational level	-.06	-.05	-.91	-.003	-.02	.09	-.02	.03	.004	.14	.06	.14	.06	.01		.01
16. Birth weight	.19*	.05	-.04	0.30	-.06	-.05	.07	.09	.04	.01	.08	.16*	-.07	.07	.04	

Note. The top part of the diagonal shows correlations in the Israeli sample and the bottom part shows correlations in the Dutch sample.
* $p<.05$ ** $p<.01$ *** $p<.001$ [a] This variable was recoded to a dichotomous variable reflecting whether infants sleep in prone or not, as this was the main difference regarding sleep position.

Table B

Correlations between parental practices related to motor development in both cultures at age 2 months, longitudinal sample.

	1	2	3	4	5	6	7	8	9	10	11	12	13	14	15	16
1. Time activity mat		-.28	.46*	.16	.12	.22	.07	-.17	-.25	-.14	-.34	.03	-.32	.16		.20
2. Time baby carrier	.04		-.15	.13	.25	.20	-.17	.05	.20	-.17	.37*	-.40*	-.09	.19		-.01
3. Time playpen	.38*	.08		.06	-.17	.24	.27	.002	-.09	-.11	-.06	.28	-.06	.11		-.02
4.Playing on the floor surface	-.01	.05	.15		-.13	.19	.00	.11	.07	.09	.05	-.24	-.03	-.20		.42*
5. Playing in the playpen	.25	.05	-.29	-.11		-.08	-.17	-.05	.13	-.11	.09	-.22	.09	.38		-.35†
6. Using a method	.20	-.03	.01	.20	.20		.13	-.06	.40*	.01	-.13	.12	.13	-.28		0.13
7. Crossing and stretching limbs	.00	-.06	-.36†	.13	.13	.26		.00	.00	-.11	-.19	.28	-.12	-.32		.12
8. Placing in sit position	-.05	.13	.13	.08	-.18	-.02	-.20		.29	.20	-.07	-.19	-.30	.07		.05
9. Placing in stand position	.24	.33†	.38*	.09	.09	.04	-.33†	.52**		-.09	-.05	-.26	-.20	-.10		.05
10. Baby swimming	.02	.05	.001	.11	.11	.05	.09	0.18	.36†		-.06	.20	.23	.00		-.43*
11. Use of sleeping bag	.12	-.28	-.05	.30	.30	.19	.34†	.02	-.04	.20		-.35†	.35†	.14		-0.25
12. Frequency of prone position	.39*	-.21	.12	.25	-.01	.32†	.25	-.36†	-.25	-.12	.30		.06	.00		-.19
13. Frequency of being held in arms	.06	.06	.14	.34	-.15	.04	-.02	-.24	.02	-.02	-.15	.38*		-.42*		-.25
14. Frequency of prone sleeping[a]	.11	.18	.14	.15	-.15	-.14	-.06	.18	.06	.15	-.07	-.16	-.07			-.35†
15. Educational level[b]	-.13	.12	-.001	.20	-.13	-.21	.22	-.22	-.22	-.20	-.24	-.09	.26	-.18		
16. Birth weight	-.20	.25	.03	-.26	.08	-.25	-.12	.02	.12	-.25	-.12	-.25	.06	-.28	.20	

Note. The top part of the diagonal shows correlations in the Israeli sample and the bottom part shows correlations in the Dutch sample.
$^{†}p<.10$ $^{*}p<.05$ $^{**}p<.01$ [a] This variable was recoded to a dichotomous variable reflecting whether infants sleep in prone or not. [b]no correlations with educational level were computed for the Israeli sample as all parents in this sample had academic level education. Given the small sample size also marginally significant correlations are reported.

Table C

Reports of parents about the setting of motor development divided in age groups

	Netherlands	Israel	*χ2(df)*	*φ*
	Age Age 1-2.5 months (N_{NL} = 33, N_{IL} = 35)			
Let infant play in the living room	95%	96%	.85(1)	.02
Let the infant play in the baby's room	7%	40%	34.82(2)***	.58
In the living room, let the infant play on:				
1. Floor surface (mat, carpet or floor)	49%	79%	10.19(1)**	.31
2. Sofa	41%	40%	.01(1)	.01
3. Playpen	88%	8%	67.57(1)***	.80
Going for walk with the stroller	77%	51%	7.91(1)**	.27
Going for a walk with the baby carrier	16%	28%	2.37(1)	.15
Going for walk with the baby in the car seat	5%	6%	NA	NA
Going for a walk, baby carried in the arms	2%	9%	NA	NA
Going for a walk without specifying how	23%	11%	NA	NA
Playing outside	16%	26%	1.75(1)	.13
Cycling	0%	0%	NA	NA
Sleeping outside	5%	4%	NA	NA
Baby swing on the playground	0%	0%	NA	NA
Swaddling during sleep	24%	25%	.02(1)	.02
Use of sleeping bag	49%	4%	26.03(1)***	.49
	Age 2.5-5.5 months (N_{NL} = 127, N_{IL} = 106)			
Let infant play in the living room	99%	100%	.70(1)	.07
Let the infant play in the baby's room	15%	40%	28.47(2)***	.44
In the living room, let the infant play on:				
1. Floor surface (mat, carpet or floor)	84%	99%	9.13(1)**	.25
2. Sofa	42%	29%	2.73(1)	.14
3. Playpen	94%	20%	85.88(1)***	.76
Going for walk with the stroller	59%	50%	1.24(1)	.09
Going for a walk with the baby carrier	17%	19%	.16(1)	.69
Going for walk with the baby in the car seat	9%	0%	NA	NA
Going for a walk, baby carried in the arms	0%	4%	NA	NA
Going for a walk without specifying how	35%	22%	NA	NA
Playing outside	8%	22%	5.73(1)*	.19
Cycling	2%	0%	NA	NA
Sleeping outside	1%	0%	NA	NA
Baby swing on the playground	0%	3%	NA	NA
Swaddling during sleep	20%	22%	.05(1)	.02
Use of sleeping bag	82%	2%	94.50(1)***	.80
	Age 5.5-8 months (N_{NL} = 29, N_{IL} = 37)			
Let infant play in the living room	20%	96%	1.96(1)	.15
Let the infant play in the baby's room	40%	39%	13.94(2)**	.39
In the living room, let the infant play on:				
1. Floor surface (mat, carpet or floor)	91%	92%	.004(1)	.01
2. Sofa	41%	19%	5.71(1)*	.25
3. Playpen	91%	19%	49.82(2)***	.73
Going for walk with the stroller	64%	43%	4.39(1)*	.22
Going for a walk with the baby carrier	22%	22%	.001(1)	.003
Going for walk with the baby in the car seat	7%	0%	NA	NA
Going for a walk, baby carried in the arms	0%	14%	NA	NA
Going for a walk without specifying how	36%	18%	NA	NA
Playing outside	9%	37%	10.15(1)**	.33
Cycling	4%	0%	NA	NA
Sleeping outside	0%	4%	NA	NA

Baby swing on the playground	0%	6%	NA	NA
Swaddling during sleep	2%	6%	.92(1)	.10
Use of sleeping bag	87%	8%	57.33(1)***	.79

Note. $^{*}p<.05$ $^{**}p<.01$ $^{***}p<.001$

Only outdoor activities that were mentioned by more than 5% of the participants in total (across both samples) were further analysed for cross-cultural differences
